# Supplementary material for: A fault-intrusion-tolerant system and deadline-aware algorithm for scheduling scientific workflow in the cloud
Source: PeerJ Comput Sci. 2021 Nov 2;7:e747. doi: 10.7717/peerj-cs.747 (PMC8576554; doi:10.7717/peerj-cs.747)
Supplement: Supplemental Information 6 [file peerj-cs-07-747-s006.zip › Myproject1.1/docs/org/cloudbus/cloudsim/DataCloudTags.html]

DataCloudTags


JavaScript is disabled on your browser.


- Overview
- Package
- Class
- Use
- Tree
- Deprecated
- Index
- Help

- Prev Class
- Next Class

- Frames
- No Frames

- All Classes

- Summary:
- Nested |
- Field |
- Constr |
- Method

- Detail:
- Field |
- Constr |
- Method


org.cloudbus.cloudsim

## Class DataCloudTags

- java.lang.Object
- - org.cloudbus.cloudsim.DataCloudTags

- ---

    

  ```
  public final class DataCloudTags
  extends java.lang.Object
  ```

  This class contains additional tags for the DataCloud functionalities, such as file information
  retrieval, file transfers, and storage info.

  Since:
  :   CloudSim Toolkit 1.0

- - ### Field Summary

    Fields

    | Modifier and Type | Field and Description |
    | --- | --- |
    | `static int` | `CTLG_ADD_MASTER` Denotes the request to register / add a master file to the Replica Catalogue.  The format of this request is Object[3] = {String filename, FileAttribute attr, Integer resID}.  The reply tag name is `CTLG_ADD_MASTER_RESULT`. |
    | `static int` | `CTLG_ADD_MASTER_ERROR` Denotes that master file addition is failed due to an unknown error |
    | `static int` | `CTLG_ADD_MASTER_ERROR_FULL` Denotes that master file addition is failed due to the catalogue is full |
    | `static int` | `CTLG_ADD_MASTER_RESULT` Sends the result of registering a master file back to sender.  The format of the reply is Object[3] = {String filename, Integer uniqueID, Integer resultID}.  NOTE: The result id is in the form of CTLG\_ADD\_MASTER\_XXXX where XXXX means the error/success message |
    | `static int` | `CTLG_ADD_MASTER_SUCCESSFUL` Denotes that master file addition is successful |
    | `static int` | `CTLG_ADD_REPLICA` Denotes the request to register / add a replica file to the Replica Catalogue.  The format of this request is Object[2] = {String lfn, Integer resourceID}.  The reply tag name is `CTLG_ADD_REPLICA_RESULT`. |
    | `static int` | `CTLG_ADD_REPLICA_ERROR` Denotes that replica file addition is failed due to an unknown error |
    | `static int` | `CTLG_ADD_REPLICA_ERROR_DOESNT_EXIST` Denotes that replica file addition is failed because the given file name does not exist in the catalogue |
    | `static int` | `CTLG_ADD_REPLICA_ERROR_FULL` Denotes that replica file addition is failed due to the catalogue is full |
    | `static int` | `CTLG_ADD_REPLICA_RESULT` Sends the result of registering a replica file back to sender.  The format of the reply is Object[2] = {String lfn, Integer resultID}.  NOTE: The result id is in the form of CTLG\_ADD\_REPLICA\_XXXX where XXXX means the error/success message |
    | `static int` | `CTLG_ADD_REPLICA_SUCCESSFUL` Denotes that replica file addition is successful |
    | `static int` | `CTLG_DELETE_MASTER` Denotes the request to de-register / delete a master file from the Replica Catalogue.  The format of this request is Object[2] = {String lfn, Integer resourceID}.  The reply tag name is `CTLG_DELETE_MASTER_RESULT`. |
    | `static int` | `CTLG_DELETE_MASTER_DOESNT_EXIST` Denotes that master file deletion is failed because the file does not exist in the catalogue |
    | `static int` | `CTLG_DELETE_MASTER_ERROR` Denotes that master file deletion is failed due to an unknown error |
    | `static int` | `CTLG_DELETE_MASTER_REPLICAS_EXIST` Denotes that master file deletion is failed because replica files are still in the catalogue. |
    | `static int` | `CTLG_DELETE_MASTER_RESULT` Sends the result of de-registering a master file back to sender.  The format of the reply is Object[2] = {String lfn, Integer resultID}.  NOTE: The result id is in the form of CTLG\_DELETE\_MASTER\_XXXX where XXXX means the error/success message |
    | `static int` | `CTLG_DELETE_MASTER_SUCCESSFUL` Denotes that master file deletion is successful |
    | `static int` | `CTLG_DELETE_REPLICA` Denotes the request to de-register / delete a replica file from the Replica Catalogue.  The format of this request is Object[2] = {String lfn, Integer resourceID}.  The reply tag name is `CTLG_DELETE_REPLICA_RESULT`. |
    | `static int` | `CTLG_DELETE_REPLICA_ERROR` Denotes that replica file deletion is failed due to an unknown error |
    | `static int` | `CTLG_DELETE_REPLICA_ERROR_DOESNT_EXIST` Denotes that replica file deletion is failed because the file does not exist in the catalogue |
    | `static int` | `CTLG_DELETE_REPLICA_RESULT` Sends the result of de-registering a replica file back to sender.  The format of the reply is Object[2] = {String lfn, Integer resultID}.  NOTE: The result id is in the form of CTLG\_DELETE\_REPLICA\_XXXX where XXXX means the error/success message |
    | `static int` | `CTLG_DELETE_REPLICA_SUCCESSFUL` Denotes that replica file deletion is successful |
    | `static int` | `CTLG_FILE_ATTR_DELIVERY` Sends the result for a file attribute back to sender.  The format of the reply is {FileAttribute fileAttr}.  NOTE: The fileAttr could be null if not found. |
    | `static int` | `CTLG_FILTER` Denotes the request to get a list of file attributes based on the given filter.  The format of this request is Object[2] = {Filter filter, Integer senderID}.  The reply tag name is `CTLG_FILTER_DELIVERY`. |
    | `static int` | `CTLG_FILTER_DELIVERY` Sends the result for a list of file attributes back to sender.  The format of the reply is {List attrList}.  NOTE: The attrList could be null if not found. |
    | `static int` | `CTLG_GET_FILE_ATTR` Denotes the request to get the attribute of a file.  The format of this request is Object[2] = {String lfn, Integer senderID}.  The reply tag name is `CTLG_FILE_ATTR_DELIVERY`. |
    | `static int` | `CTLG_GET_REPLICA` Denotes the request for a location of a replica file.  The format of this request is Object[2] = {String lfn, Integer senderID}.  The reply tag name is `CTLG_REPLICA_DELIVERY`.  NOTE: This request only ask for one location only not all. |
    | `static int` | `CTLG_GET_REPLICA_LIST` Denotes the request for all locations of a replica file.  The format of this request is Object[2] = {String lfn, Integer senderID}.  The reply tag name is `CTLG_REPLICA_LIST_DELIVERY`. |
    | `static int` | `CTLG_MODIFY_MASTER` Denotes the request to modify an existing master file information stored in the Replica Catalogue.  The format of this request is Object[3] = {String filename, FileAttribute attr, Integer resID}.  The reply tag name is `CTLG_MODIFY_MASTER_RESULT`. |
    | `static int` | `CTLG_MODIFY_MASTER_ERROR` Denotes that master file modification is failed due to an unknown error |
    | `static int` | `CTLG_MODIFY_MASTER_ERROR_DOESNT_EXIST` Denotes that master file modification is failed because the file does not exist in the catalogue |
    | `static int` | `CTLG_MODIFY_MASTER_ERROR_READ_ONLY` Denotes that master file modification is failed because the file attribute is set to a read-only |
    | `static int` | `CTLG_MODIFY_MASTER_RESULT` Sends the result of modifying a master file back to sender.  The format of the reply is Object[2] = {String lfn, Integer resultID}.  NOTE: The result id is in the form of CTLG\_MODIFY\_MASTER\_XXXX where XXXX means the error/success message |
    | `static int` | `CTLG_MODIFY_MASTER_SUCCESSFUL` Denotes that master file deletion is successful |
    | `static int` | `CTLG_REPLICA_DELIVERY` Sends the result for a location of a replica file back to sender.  The format of the reply is Object[2] = {String lfn, Integer resourceID}.  NOTE: The resourceID could be -1 if not found. |
    | `static int` | `CTLG_REPLICA_LIST_DELIVERY` Sends the result for all locations of a replica file back to sender.  The format of the reply is Object[2] = {String lfn, List locationList}.  NOTE: The locationList could be null if not found. |
    | `static int` | `DATAcloudlet_SUBMIT` Denotes the submission of a DataCloudlet. |
    | `static int` | `DEFAULT_MTU` Default Maximum Transmission Unit (MTU) of a link in bytes |
    | `static int` | `DEFAULT_STORAGE_SIZE` The default storage size (10 GByte) |
    | `static int` | `FILE_ADD_ERROR` Denotes that file addition is failed due to an unknown error |
    | `static int` | `FILE_ADD_ERROR_ACCESS_DENIED` Denotes that file addition is failed because access/permission denied or not authorized |
    | `static int` | `FILE_ADD_ERROR_EMPTY` Denotes that file addition is failed because the given file is empty |
    | `static int` | `FILE_ADD_ERROR_EXIST_READ_ONLY` Denotes that file addition is failed because the file already exists in the catalogue and it is read-only file |
    | `static int` | `FILE_ADD_ERROR_STORAGE_FULL` Denotes that file addition is failed because the storage is full |
    | `static int` | `FILE_ADD_MASTER` Requests for a master file to be added to the local storage(s).  The format of this request is Object[2] = {File obj, Integer senderID}.  The reply tag name is `FILE_ADD_MASTER_RESULT`. |
    | `static int` | `FILE_ADD_MASTER_RESULT` Sends the result of adding a master file back to sender.  The format of the reply is Object[3] = {String lfn, Integer uniqueID, Integer resultID}.  NOTE: The result id is in the form of FILE\_ADD\_XXXX where XXXX means the error/success message |
    | `static int` | `FILE_ADD_REPLICA` Requests for a replica file to be added from the local storage(s).  The format of this request is Object[2] = {File obj, Integer senderID}.  The reply tag name is `FILE_ADD_REPLICA_RESULT`. |
    | `static int` | `FILE_ADD_REPLICA_RESULT` Sends the result of adding a replica file back to sender.  The format of the reply is Object[2] = {String lfn, Integer resultID}.  NOTE: The result id is in the form of FILE\_ADD\_XXXX where XXXX means the error/success message |
    | `static int` | `FILE_ADD_SUCCESSFUL` Denotes that file addition is successful |
    | `static int` | `FILE_DELETE_ERROR` Denotes that file deletion is failed due to an unknown error |
    | `static int` | `FILE_DELETE_ERROR_ACCESS_DENIED` Denotes that file deletion is failed because access/permission denied or not authorized |
    | `static int` | `FILE_DELETE_ERROR_DOESNT_EXIST` Denotes that file deletion is failed because the file does not exist in the storage nor catalogue |
    | `static int` | `FILE_DELETE_ERROR_IN_USE` Denotes that file deletion is failed because it is currently used by others |
    | `static int` | `FILE_DELETE_ERROR_READ_ONLY` Denotes that file deletion is failed because it is a read-only file |
    | `static int` | `FILE_DELETE_MASTER` Requests for a master file to be deleted from the local storage(s).  The format of this request is Object[2] = {String lfn, Integer senderID}.  The reply tag name is `FILE_DELETE_MASTER_RESULT`. |
    | `static int` | `FILE_DELETE_MASTER_RESULT` Sends the result of deleting a master file back to sender.  The format of the reply is Object[2] = {String lfn, Integer resultID}.  NOTE: The result id is in the form of FILE\_DELETE\_XXXX where XXXX means the error/success message |
    | `static int` | `FILE_DELETE_REPLICA` Requests for a replica file to be deleted from the local storage(s).  The format of this request is Object[2] = {String lfn, Integer senderID}.  The reply tag name is `FILE_DELETE_REPLICA_RESULT`. |
    | `static int` | `FILE_DELETE_REPLICA_RESULT` Sends the result of deleting a replica file back to sender.  The format of the reply is Object[2] = {String lfn, Integer resultID}.  NOTE: The result id is in the form of FILE\_DELETE\_XXXX where XXXX means the error/success message |
    | `static int` | `FILE_DELETE_SUCCESSFUL` Denotes that file deletion is successful |
    | `static int` | `FILE_DELIVERY` Sends the file to the requester. |
    | `static int` | `FILE_MODIFY` Requests for a file to be modified from the local storage(s).  The format of this request is Object[2] = {File obj, Integer senderID}.  The reply tag name is `FILE_MODIFY_RESULT`. |
    | `static int` | `FILE_MODIFY_ERROR` Denotes that file modification is failed due to an unknown error |
    | `static int` | `FILE_MODIFY_ERROR_ACCESS_DENIED` Denotes that file modification is failed because access/permission denied or not authorized |
    | `static int` | `FILE_MODIFY_ERROR_DOESNT_EXIST` Denotes that file modification is failed because the file does not exist |
    | `static int` | `FILE_MODIFY_ERROR_IN_USE` Denotes that file modification is failed because the file is currently used by others |
    | `static int` | `FILE_MODIFY_ERROR_READ_ONLY` Denotes that file modification is failed because it is a read-only file |
    | `static int` | `FILE_MODIFY_RESULT` Sends the result of deleting a file back to sender.  The format of the reply is Object[2] = {String lfn, Integer resultID}.  NOTE: The result id is in the form of FILE\_MODIFY\_XXXX where XXXX means the error/success message |
    | `static int` | `FILE_MODIFY_SUCCESSFUL` Denotes that file modification is successful |
    | `static int` | `FILE_REQUEST` Requests for a file that is stored on the local storage(s).  The format of this request is Object[2] = {String lfn, Integer senderID}.  The reply tag name is `FILE_DELIVERY`. |
    | `static int` | `INQUIRY_GLOBAL_RC_LIST` Denotes a list of Replica Catalogue (RC) entities that are listed in other regional Data GIS entities. |
    | `static int` | `INQUIRY_LOCAL_RC_LIST` Denotes a list of all Replica Catalogue (RC) entities that are listed in this regional Data GIS entity. |
    | `static int` | `INQUIRY_RC_LIST` Denotes a list of Replica Catalogue IDs. |
    | `static int` | `INQUIRY_RC_RESULT` Denotes a result regarding to a list of Replica Catalogue IDs. |
    | `static int` | `PKT_SIZE` The default packet size (in byte) for sending events to other entity. |
    | `static int` | `REGISTER_REPLICA_CTLG` Registers a Replica Catalogue (RC) entity to a Data GIS |
  - ### Method Summary

    - ### Methods inherited from class java.lang.Object

      `clone, equals, finalize, getClass, hashCode, notify, notifyAll, toString, wait, wait, wait`

- - ### Field Detail


    - #### DEFAULT\_MTU

      ```
      public static final int DEFAULT_MTU
      ```

      Default Maximum Transmission Unit (MTU) of a link in bytes

      See Also:
      :   Constant Field Values


    - #### PKT\_SIZE

      ```
      public static final int PKT_SIZE
      ```

      The default packet size (in byte) for sending events to other entity.

      See Also:
      :   Constant Field Values


    - #### DEFAULT\_STORAGE\_SIZE

      ```
      public static final int DEFAULT_STORAGE_SIZE
      ```

      The default storage size (10 GByte)

      See Also:
      :   Constant Field Values


    - #### REGISTER\_REPLICA\_CTLG

      ```
      public static final int REGISTER_REPLICA_CTLG
      ```

      Registers a Replica Catalogue (RC) entity to a Data GIS

      See Also:
      :   Constant Field Values


    - #### INQUIRY\_LOCAL\_RC\_LIST

      ```
      public static final int INQUIRY_LOCAL_RC_LIST
      ```

      Denotes a list of all Replica Catalogue (RC) entities that are listed in this regional Data
      GIS entity. This tag should be called from a user to Data GIS.

      See Also:
      :   Constant Field Values


    - #### INQUIRY\_GLOBAL\_RC\_LIST

      ```
      public static final int INQUIRY_GLOBAL_RC_LIST
      ```

      Denotes a list of Replica Catalogue (RC) entities that are listed in other regional Data GIS
      entities. This tag should be called from a user to Data GIS.

      See Also:
      :   Constant Field Values


    - #### INQUIRY\_RC\_LIST

      ```
      public static final int INQUIRY_RC_LIST
      ```

      Denotes a list of Replica Catalogue IDs. This tag should be called from a Regional Data GIS
      to another

      See Also:
      :   Constant Field Values


    - #### INQUIRY\_RC\_RESULT

      ```
      public static final int INQUIRY_RC_RESULT
      ```

      Denotes a result regarding to a list of Replica Catalogue IDs. This tag should be called from
      a Regional Data GIS to a sender Regional Data GIS.

      See Also:
      :   Constant Field Values


    - #### DATAcloudlet\_SUBMIT

      ```
      public static final int DATAcloudlet_SUBMIT
      ```

      Denotes the submission of a DataCloudlet. This tag is normally used between user and
      DataCloudResource entity.

      See Also:
      :   Constant Field Values


    - #### FILE\_REQUEST

      ```
      public static final int FILE_REQUEST
      ```

      Requests for a file that is stored on the local storage(s).  
      The format of this request is Object[2] = {String lfn, Integer senderID}.  
      The reply tag name is `FILE_DELIVERY`.

      See Also:
      :   Constant Field Values


    - #### FILE\_DELIVERY

      ```
      public static final int FILE_DELIVERY
      ```

      Sends the file to the requester. The format of the reply is File or null if error happens

      See Also:
      :   Constant Field Values


    - #### FILE\_ADD\_MASTER

      ```
      public static final int FILE_ADD_MASTER
      ```

      Requests for a master file to be added to the local storage(s).  
      The format of this request is Object[2] = {File obj, Integer senderID}.  
      The reply tag name is `FILE_ADD_MASTER_RESULT`.

      See Also:
      :   Constant Field Values


    - #### FILE\_ADD\_MASTER\_RESULT

      ```
      public static final int FILE_ADD_MASTER_RESULT
      ```

      Sends the result of adding a master file back to sender.  
      The format of the reply is Object[3] = {String lfn, Integer uniqueID, Integer resultID}.  
      NOTE: The result id is in the form of FILE\_ADD\_XXXX where XXXX means the error/success
      message

      See Also:
      :   Constant Field Values


    - #### FILE\_ADD\_REPLICA

      ```
      public static final int FILE_ADD_REPLICA
      ```

      Requests for a replica file to be added from the local storage(s).  
      The format of this request is Object[2] = {File obj, Integer senderID}.  
      The reply tag name is `FILE_ADD_REPLICA_RESULT`.

      See Also:
      :   Constant Field Values


    - #### FILE\_ADD\_REPLICA\_RESULT

      ```
      public static final int FILE_ADD_REPLICA_RESULT
      ```

      Sends the result of adding a replica file back to sender.  
      The format of the reply is Object[2] = {String lfn, Integer resultID}.  
      NOTE: The result id is in the form of FILE\_ADD\_XXXX where XXXX means the error/success
      message

      See Also:
      :   Constant Field Values


    - #### FILE\_ADD\_SUCCESSFUL

      ```
      public static final int FILE_ADD_SUCCESSFUL
      ```

      Denotes that file addition is successful

      See Also:
      :   Constant Field Values


    - #### FILE\_ADD\_ERROR\_STORAGE\_FULL

      ```
      public static final int FILE_ADD_ERROR_STORAGE_FULL
      ```

      Denotes that file addition is failed because the storage is full

      See Also:
      :   Constant Field Values


    - #### FILE\_ADD\_ERROR\_EMPTY

      ```
      public static final int FILE_ADD_ERROR_EMPTY
      ```

      Denotes that file addition is failed because the given file is empty

      See Also:
      :   Constant Field Values


    - #### FILE\_ADD\_ERROR\_EXIST\_READ\_ONLY

      ```
      public static final int FILE_ADD_ERROR_EXIST_READ_ONLY
      ```

      Denotes that file addition is failed because the file already exists in the catalogue and it
      is read-only file

      See Also:
      :   Constant Field Values


    - #### FILE\_ADD\_ERROR

      ```
      public static final int FILE_ADD_ERROR
      ```

      Denotes that file addition is failed due to an unknown error

      See Also:
      :   Constant Field Values


    - #### FILE\_ADD\_ERROR\_ACCESS\_DENIED

      ```
      public static final int FILE_ADD_ERROR_ACCESS_DENIED
      ```

      Denotes that file addition is failed because access/permission denied or not authorized

      See Also:
      :   Constant Field Values


    - #### FILE\_DELETE\_MASTER

      ```
      public static final int FILE_DELETE_MASTER
      ```

      Requests for a master file to be deleted from the local storage(s).  
      The format of this request is Object[2] = {String lfn, Integer senderID}.  
      The reply tag name is `FILE_DELETE_MASTER_RESULT`.

      See Also:
      :   Constant Field Values


    - #### FILE\_DELETE\_MASTER\_RESULT

      ```
      public static final int FILE_DELETE_MASTER_RESULT
      ```

      Sends the result of deleting a master file back to sender.  
      The format of the reply is Object[2] = {String lfn, Integer resultID}.  
      NOTE: The result id is in the form of FILE\_DELETE\_XXXX where XXXX means the error/success
      message

      See Also:
      :   Constant Field Values


    - #### FILE\_DELETE\_REPLICA

      ```
      public static final int FILE_DELETE_REPLICA
      ```

      Requests for a replica file to be deleted from the local storage(s).  
      The format of this request is Object[2] = {String lfn, Integer senderID}.  
      The reply tag name is `FILE_DELETE_REPLICA_RESULT`.

      See Also:
      :   Constant Field Values


    - #### FILE\_DELETE\_REPLICA\_RESULT

      ```
      public static final int FILE_DELETE_REPLICA_RESULT
      ```

      Sends the result of deleting a replica file back to sender.  
      The format of the reply is Object[2] = {String lfn, Integer resultID}.  
      NOTE: The result id is in the form of FILE\_DELETE\_XXXX where XXXX means the error/success
      message

      See Also:
      :   Constant Field Values


    - #### FILE\_DELETE\_SUCCESSFUL

      ```
      public static final int FILE_DELETE_SUCCESSFUL
      ```

      Denotes that file deletion is successful

      See Also:
      :   Constant Field Values


    - #### FILE\_DELETE\_ERROR

      ```
      public static final int FILE_DELETE_ERROR
      ```

      Denotes that file deletion is failed due to an unknown error

      See Also:
      :   Constant Field Values


    - #### FILE\_DELETE\_ERROR\_READ\_ONLY

      ```
      public static final int FILE_DELETE_ERROR_READ_ONLY
      ```

      Denotes that file deletion is failed because it is a read-only file

      See Also:
      :   Constant Field Values


    - #### FILE\_DELETE\_ERROR\_DOESNT\_EXIST

      ```
      public static final int FILE_DELETE_ERROR_DOESNT_EXIST
      ```

      Denotes that file deletion is failed because the file does not exist in the storage nor
      catalogue

      See Also:
      :   Constant Field Values


    - #### FILE\_DELETE\_ERROR\_IN\_USE

      ```
      public static final int FILE_DELETE_ERROR_IN_USE
      ```

      Denotes that file deletion is failed because it is currently used by others

      See Also:
      :   Constant Field Values


    - #### FILE\_DELETE\_ERROR\_ACCESS\_DENIED

      ```
      public static final int FILE_DELETE_ERROR_ACCESS_DENIED
      ```

      Denotes that file deletion is failed because access/permission denied or not authorized

      See Also:
      :   Constant Field Values


    - #### FILE\_MODIFY

      ```
      public static final int FILE_MODIFY
      ```

      Requests for a file to be modified from the local storage(s).  
      The format of this request is Object[2] = {File obj, Integer senderID}.  
      The reply tag name is `FILE_MODIFY_RESULT`.

      See Also:
      :   Constant Field Values


    - #### FILE\_MODIFY\_RESULT

      ```
      public static final int FILE_MODIFY_RESULT
      ```

      Sends the result of deleting a file back to sender.  
      The format of the reply is Object[2] = {String lfn, Integer resultID}.  
      NOTE: The result id is in the form of FILE\_MODIFY\_XXXX where XXXX means the error/success
      message

      See Also:
      :   Constant Field Values


    - #### FILE\_MODIFY\_SUCCESSFUL

      ```
      public static final int FILE_MODIFY_SUCCESSFUL
      ```

      Denotes that file modification is successful

      See Also:
      :   Constant Field Values


    - #### FILE\_MODIFY\_ERROR

      ```
      public static final int FILE_MODIFY_ERROR
      ```

      Denotes that file modification is failed due to an unknown error

      See Also:
      :   Constant Field Values


    - #### FILE\_MODIFY\_ERROR\_READ\_ONLY

      ```
      public static final int FILE_MODIFY_ERROR_READ_ONLY
      ```

      Denotes that file modification is failed because it is a read-only file

      See Also:
      :   Constant Field Values


    - #### FILE\_MODIFY\_ERROR\_DOESNT\_EXIST

      ```
      public static final int FILE_MODIFY_ERROR_DOESNT_EXIST
      ```

      Denotes that file modification is failed because the file does not exist

      See Also:
      :   Constant Field Values


    - #### FILE\_MODIFY\_ERROR\_IN\_USE

      ```
      public static final int FILE_MODIFY_ERROR_IN_USE
      ```

      Denotes that file modification is failed because the file is currently used by others

      See Also:
      :   Constant Field Values


    - #### FILE\_MODIFY\_ERROR\_ACCESS\_DENIED

      ```
      public static final int FILE_MODIFY_ERROR_ACCESS_DENIED
      ```

      Denotes that file modification is failed because access/permission denied or not authorized

      See Also:
      :   Constant Field Values


    - #### CTLG\_GET\_REPLICA

      ```
      public static final int CTLG_GET_REPLICA
      ```

      Denotes the request for a location of a replica file.  
      The format of this request is Object[2] = {String lfn, Integer senderID}.  
      The reply tag name is `CTLG_REPLICA_DELIVERY`.  
      NOTE: This request only ask for one location only not all.

      See Also:
      :   Constant Field Values


    - #### CTLG\_REPLICA\_DELIVERY

      ```
      public static final int CTLG_REPLICA_DELIVERY
      ```

      Sends the result for a location of a replica file back to sender.  
      The format of the reply is Object[2] = {String lfn, Integer resourceID}.  
      NOTE: The resourceID could be -1 if not found.

      See Also:
      :   Constant Field Values


    - #### CTLG\_GET\_REPLICA\_LIST

      ```
      public static final int CTLG_GET_REPLICA_LIST
      ```

      Denotes the request for all locations of a replica file.  
      The format of this request is Object[2] = {String lfn, Integer senderID}.  
      The reply tag name is `CTLG_REPLICA_LIST_DELIVERY`.

      See Also:
      :   Constant Field Values


    - #### CTLG\_REPLICA\_LIST\_DELIVERY

      ```
      public static final int CTLG_REPLICA_LIST_DELIVERY
      ```

      Sends the result for all locations of a replica file back to sender.  
      The format of the reply is Object[2] = {String lfn, List locationList}.  
      NOTE: The locationList could be null if not found.

      See Also:
      :   Constant Field Values


    - #### CTLG\_GET\_FILE\_ATTR

      ```
      public static final int CTLG_GET_FILE_ATTR
      ```

      Denotes the request to get the attribute of a file.  
      The format of this request is Object[2] = {String lfn, Integer senderID}.  
      The reply tag name is `CTLG_FILE_ATTR_DELIVERY`.

      See Also:
      :   Constant Field Values


    - #### CTLG\_FILE\_ATTR\_DELIVERY

      ```
      public static final int CTLG_FILE_ATTR_DELIVERY
      ```

      Sends the result for a file attribute back to sender.  
      The format of the reply is {FileAttribute fileAttr}.  
      NOTE: The fileAttr could be null if not found.

      See Also:
      :   Constant Field Values


    - #### CTLG\_FILTER

      ```
      public static final int CTLG_FILTER
      ```

      Denotes the request to get a list of file attributes based on the given filter.  
      The format of this request is Object[2] = {Filter filter, Integer senderID}.  
      The reply tag name is `CTLG_FILTER_DELIVERY`.

      See Also:
      :   Constant Field Values


    - #### CTLG\_FILTER\_DELIVERY

      ```
      public static final int CTLG_FILTER_DELIVERY
      ```

      Sends the result for a list of file attributes back to sender.  
      The format of the reply is {List attrList}.  
      NOTE: The attrList could be null if not found.

      See Also:
      :   Constant Field Values


    - #### CTLG\_ADD\_MASTER

      ```
      public static final int CTLG_ADD_MASTER
      ```

      Denotes the request to register / add a master file to the Replica Catalogue.  
      The format of this request is Object[3] = {String filename, FileAttribute attr, Integer
      resID}.  
      The reply tag name is `CTLG_ADD_MASTER_RESULT`.

      See Also:
      :   Constant Field Values


    - #### CTLG\_ADD\_MASTER\_RESULT

      ```
      public static final int CTLG_ADD_MASTER_RESULT
      ```

      Sends the result of registering a master file back to sender.  
      The format of the reply is Object[3] = {String filename, Integer uniqueID, Integer resultID}.  
      NOTE: The result id is in the form of CTLG\_ADD\_MASTER\_XXXX where XXXX means the error/success
      message

      See Also:
      :   Constant Field Values


    - #### CTLG\_ADD\_MASTER\_SUCCESSFUL

      ```
      public static final int CTLG_ADD_MASTER_SUCCESSFUL
      ```

      Denotes that master file addition is successful

      See Also:
      :   Constant Field Values


    - #### CTLG\_ADD\_MASTER\_ERROR

      ```
      public static final int CTLG_ADD_MASTER_ERROR
      ```

      Denotes that master file addition is failed due to an unknown error

      See Also:
      :   Constant Field Values


    - #### CTLG\_ADD\_MASTER\_ERROR\_FULL

      ```
      public static final int CTLG_ADD_MASTER_ERROR_FULL
      ```

      Denotes that master file addition is failed due to the catalogue is full

      See Also:
      :   Constant Field Values


    - #### CTLG\_DELETE\_MASTER

      ```
      public static final int CTLG_DELETE_MASTER
      ```

      Denotes the request to de-register / delete a master file from the Replica Catalogue.  
      The format of this request is Object[2] = {String lfn, Integer resourceID}.  
      The reply tag name is `CTLG_DELETE_MASTER_RESULT`.

      See Also:
      :   Constant Field Values


    - #### CTLG\_DELETE\_MASTER\_RESULT

      ```
      public static final int CTLG_DELETE_MASTER_RESULT
      ```

      Sends the result of de-registering a master file back to sender.  
      The format of the reply is Object[2] = {String lfn, Integer resultID}.  
      NOTE: The result id is in the form of CTLG\_DELETE\_MASTER\_XXXX where XXXX means the
      error/success message

      See Also:
      :   Constant Field Values


    - #### CTLG\_DELETE\_MASTER\_SUCCESSFUL

      ```
      public static final int CTLG_DELETE_MASTER_SUCCESSFUL
      ```

      Denotes that master file deletion is successful

      See Also:
      :   Constant Field Values


    - #### CTLG\_DELETE\_MASTER\_ERROR

      ```
      public static final int CTLG_DELETE_MASTER_ERROR
      ```

      Denotes that master file deletion is failed due to an unknown error

      See Also:
      :   Constant Field Values


    - #### CTLG\_DELETE\_MASTER\_DOESNT\_EXIST

      ```
      public static final int CTLG_DELETE_MASTER_DOESNT_EXIST
      ```

      Denotes that master file deletion is failed because the file does not exist in the catalogue

      See Also:
      :   Constant Field Values


    - #### CTLG\_DELETE\_MASTER\_REPLICAS\_EXIST

      ```
      public static final int CTLG_DELETE_MASTER_REPLICAS_EXIST
      ```

      Denotes that master file deletion is failed because replica files are still in the catalogue.
      All replicas need to be deleted first.

      See Also:
      :   Constant Field Values


    - #### CTLG\_ADD\_REPLICA

      ```
      public static final int CTLG_ADD_REPLICA
      ```

      Denotes the request to register / add a replica file to the Replica Catalogue.  
      The format of this request is Object[2] = {String lfn, Integer resourceID}.  
      The reply tag name is `CTLG_ADD_REPLICA_RESULT`.

      See Also:
      :   Constant Field Values


    - #### CTLG\_ADD\_REPLICA\_RESULT

      ```
      public static final int CTLG_ADD_REPLICA_RESULT
      ```

      Sends the result of registering a replica file back to sender.  
      The format of the reply is Object[2] = {String lfn, Integer resultID}.  
      NOTE: The result id is in the form of CTLG\_ADD\_REPLICA\_XXXX where XXXX means the
      error/success message

      See Also:
      :   Constant Field Values


    - #### CTLG\_ADD\_REPLICA\_SUCCESSFUL

      ```
      public static final int CTLG_ADD_REPLICA_SUCCESSFUL
      ```

      Denotes that replica file addition is successful

      See Also:
      :   Constant Field Values


    - #### CTLG\_ADD\_REPLICA\_ERROR

      ```
      public static final int CTLG_ADD_REPLICA_ERROR
      ```

      Denotes that replica file addition is failed due to an unknown error

      See Also:
      :   Constant Field Values


    - #### CTLG\_ADD\_REPLICA\_ERROR\_DOESNT\_EXIST

      ```
      public static final int CTLG_ADD_REPLICA_ERROR_DOESNT_EXIST
      ```

      Denotes that replica file addition is failed because the given file name does not exist in
      the catalogue

      See Also:
      :   Constant Field Values


    - #### CTLG\_ADD\_REPLICA\_ERROR\_FULL

      ```
      public static final int CTLG_ADD_REPLICA_ERROR_FULL
      ```

      Denotes that replica file addition is failed due to the catalogue is full

      See Also:
      :   Constant Field Values


    - #### CTLG\_DELETE\_REPLICA

      ```
      public static final int CTLG_DELETE_REPLICA
      ```

      Denotes the request to de-register / delete a replica file from the Replica Catalogue.  
      The format of this request is Object[2] = {String lfn, Integer resourceID}.  
      The reply tag name is `CTLG_DELETE_REPLICA_RESULT`.

      See Also:
      :   Constant Field Values


    - #### CTLG\_DELETE\_REPLICA\_RESULT

      ```
      public static final int CTLG_DELETE_REPLICA_RESULT
      ```

      Sends the result of de-registering a replica file back to sender.  
      The format of the reply is Object[2] = {String lfn, Integer resultID}.  
      NOTE: The result id is in the form of CTLG\_DELETE\_REPLICA\_XXXX where XXXX means the
      error/success message

      See Also:
      :   Constant Field Values


    - #### CTLG\_DELETE\_REPLICA\_SUCCESSFUL

      ```
      public static final int CTLG_DELETE_REPLICA_SUCCESSFUL
      ```

      Denotes that replica file deletion is successful

      See Also:
      :   Constant Field Values


    - #### CTLG\_DELETE\_REPLICA\_ERROR

      ```
      public static final int CTLG_DELETE_REPLICA_ERROR
      ```

      Denotes that replica file deletion is failed due to an unknown error

      See Also:
      :   Constant Field Values


    - #### CTLG\_DELETE\_REPLICA\_ERROR\_DOESNT\_EXIST

      ```
      public static final int CTLG_DELETE_REPLICA_ERROR_DOESNT_EXIST
      ```

      Denotes that replica file deletion is failed because the file does not exist in the catalogue

      See Also:
      :   Constant Field Values


    - #### CTLG\_MODIFY\_MASTER

      ```
      public static final int CTLG_MODIFY_MASTER
      ```

      Denotes the request to modify an existing master file information stored in the Replica
      Catalogue.  
      The format of this request is Object[3] = {String filename, FileAttribute attr, Integer
      resID}.  
      The reply tag name is `CTLG_MODIFY_MASTER_RESULT`.

      See Also:
      :   Constant Field Values


    - #### CTLG\_MODIFY\_MASTER\_RESULT

      ```
      public static final int CTLG_MODIFY_MASTER_RESULT
      ```

      Sends the result of modifying a master file back to sender.  
      The format of the reply is Object[2] = {String lfn, Integer resultID}.  
      NOTE: The result id is in the form of CTLG\_MODIFY\_MASTER\_XXXX where XXXX means the
      error/success message

      See Also:
      :   Constant Field Values


    - #### CTLG\_MODIFY\_MASTER\_SUCCESSFUL

      ```
      public static final int CTLG_MODIFY_MASTER_SUCCESSFUL
      ```

      Denotes that master file deletion is successful

      See Also:
      :   Constant Field Values


    - #### CTLG\_MODIFY\_MASTER\_ERROR

      ```
      public static final int CTLG_MODIFY_MASTER_ERROR
      ```

      Denotes that master file modification is failed due to an unknown error

      See Also:
      :   Constant Field Values


    - #### CTLG\_MODIFY\_MASTER\_ERROR\_DOESNT\_EXIST

      ```
      public static final int CTLG_MODIFY_MASTER_ERROR_DOESNT_EXIST
      ```

      Denotes that master file modification is failed because the file does not exist in the
      catalogue

      See Also:
      :   Constant Field Values


    - #### CTLG\_MODIFY\_MASTER\_ERROR\_READ\_ONLY

      ```
      public static final int CTLG_MODIFY_MASTER_ERROR_READ_ONLY
      ```

      Denotes that master file modification is failed because the file attribute is set to a
      read-only

      See Also:
      :   Constant Field Values


- Overview
- Package
- Class
- Use
- Tree
- Deprecated
- Index
- Help

- Prev Class
- Next Class

- Frames
- No Frames

- All Classes

- Summary:
- Nested |
- Field |
- Constr |
- Method

- Detail:
- Field |
- Constr |
- Method
